# Autophagy-Related LC3 Accumulation Interacted Directly With LIR Containing RIPK1 and RIPK3, Stimulating Necroptosis in Hypoxic Cardiomyocytes

**DOI:** 10.3389/fcell.2021.679637

**Published:** 2021-07-23

**Authors:** Yao Huang, Yanhai Feng, Lin Cui, Lei Yang, Qiong Zhang, Junhui Zhang, Xupin Jiang, Xingyue Zhang, Yanling Lv, Jie-Zhi Jia, Dong-Xia Zhang, Yue-Sheng Huang

**Affiliations:** ^1^State Key Laboratory of Trauma, Burns and Combined Injury, Institute of Burn Research, Southwest Hospital, Army Medical University (Third Military Medical University), Chongqing, China; ^2^Department of Wound Repair, and Institute of Wound Repair, Shenzhen People’s Hospital (The Second Clinical Medical College, Jinan University; The First Affiliated Hospital, Southern University of Science and Technology), Shenzhen, China

**Keywords:** myocardial hypoxia, necroptosis, autophagy, autophagosome, LC3

## Abstract

The exact relationships and detailed mechanisms between autophagy and necroptosis remain obscure. Here, we demonstrated the link between accumulated autophagosome and necroptosis by intervening with autophagic flux. We first confirmed that the LC3 interacting region (LIR) domain is present in the protein sequences of RIPK1 and RIPK3. Mutual effects among LC3, RIPK1, and RIPK3 have been identified in myocardium and cardiomyocytes. Direct LC3-RIPK1 and LC3-RIPK3 interactions were confirmed by pull-down assays, and their interactions were deleted after LIR domain mutation. Moreover, after disrupting autophagic flux under normoxia with bafilomycin A1 treatment, or with LC3 or ATG5 overexpression adenovirus, RIPK1, RIPK3, p-RIPK3, and p-MLKL levels increased, suggesting necroptosis activation. Severe disruptions in autophagic flux were observed under hypoxia and bafilomycin A1 co-treated cardiomyocytes and myocardium and led to more significant activation of necroptosis. Conversely, after alleviating hypoxia-induced autophagic flux impairment with LC3 or ATG5 knockdown adenovirus, the effects of hypoxia on RIPK1 and RIPK3 levels were reduced, which resulted in decreased p-RIPK3 and p-MLKL. Furthermore, necroptosis was inhibited by siRNAs against RIPK1 and RIPK3 under hypoxia or normoxia. Based on our results, LIR domain mediated LC3-RIPK1 and LC3-RIPK3 interaction. Besides, autophagosome accumulation under hypoxia lead to necrosome formation and, in turn, necroptosis, while when autophagic flux was uninterrupted, RIPK1 and RIPK3 were cleared through an autophagy-related pathway which inhibited necroptosis. These findings provide novel insights for the role of LC3 in regulating cardiomyocyte necroptosis, indicating its therapeutic potential in the prevention and treatment of hypoxic myocardial injury and other hypoxia-related diseases.

## Introduction

Heart, an essential component of circulation system, plays an important role in maintaining life activity. The myocardium is exposed to hypoxic injury under several conditions, including long-term residence in plateau environments, coronary heart disease, traumatic hemorrhage, organ transplantation, pulmonary disease, pulmonary embolism, aspiration, cardiac arrest, and severe burns (Davies and Wedzicha, 1993; Cui et al., 2020). Therefore, a better understanding of the mechanisms underlying myocardial injury caused by hypoxia may yield new therapeutic targets against these pathological conditions.

Hypoxia has been reported to induce necroptosis in several pathological conditions. For example, the pigment epithelium-derived factor (PEDF) is shown to activate necroptosis by regulating RIPK3 in hypoxic cardiomyocytes; however, the detailed mechanisms of hypoxia-induced necrosome formation are still poorly understood (Gao et al., 2015). Necroptosis is a form of regulated necrosis. Receptor-interacting protein kinase (RIPK) 1 (RIPK1, also known as RIP1) was the first discovered molecule in the necroptosis pathway and has been identified as a component of the death complex (i.e., necrosome) (Lee et al., 2004; Mompeán et al., 2018). Upon inhibition of the Fas-associated death domain (FADD) or caspase activity by genetic or chemical methods, RIPK1 and RIPK3 (also known as RIP3) form the necrosome through the homotypic interaction motif (RHIM) domains. The necrosome phosphorylates RIPK3 at S227 (S232 for mouse RIPK3), and then the mixed lineage kinase domain-like pseudokinase (MLKL) is activated by phosphorylation at S358 (S345 for mouse MLKL) (Sun et al., 2012). The phosphorylated MLKL (p-MLKL) serves as a marker of necroptosis since p-MLKL transport to the plasma membrane is linked to cell death (Zhang et al., 2016). Therefore, the necrosome is a key regulator of necroptosis. Thus, determining the mechanisms underlying necrosome formation in hypoxic heart is important in preventing myocardial injury caused by hypoxia.

Autophagy, which we used here to refer to macroautophagy, is a highly regulated process involved in the degradation of proteins and damaged organelles through the lysosomal system (Mizumura et al., 2018). The initiation of autophagy is indicated by the development of a double-layered, crescent-shaped membrane known as a phagophore (Tanida et al., 2008). It elongates and matures into an autophagosome, accompanied by the conversion of the microtubule-associated protein 1A/1B-light chain 3 (LC3)-I to LC3-II. The autophagosomes sequester and engulf various proteins and damaged organelles, and then degrade them in lysosomes (Parzych and Klionsky, 2014). However, our previous studies have demonstrated that autophagosomes cannot be degraded in hypoxic cardiomyocytes (Cui et al., 2020). A number of studies have reported a possible link between autophagy and necroptosis; for instance, ischemic stroke leads to neuronal and astrocytic cell death *via* RIPK1 when autophagy is activated (Ni and Gu, 2018). On the other hand, suppressed autophagic flux contributes to cardiomyocyte death by activating necroptotic pathways (Ogasawara et al., 2017). Some studies have suggested that impaired autophagy promotes necroptosis by increasing the levels of reactive oxygen species (Zhang et al., 2017). These contradictory results may be caused by different research conditions and models. To date, the specific mechanisms of autophagy-regulated necroptosis remain unclear.

Therefore, in this study, in order to define the effects of autophagy on necroptosis in hypoxic cardiomyocytes, we exposed the cells or the mice to hypoxia and evaluated the relationships between LC3 and RIPK1/3. We found that LC3 interacts directly with RIPK1 and RIPK3 *via* LIR domain to regulate necroptosis in cardiomyocytes. The findings we present here provide a novel insight into the role of autophagic-related LC3 in regulating cardiomyocyte necroptosis, indicating that therapeutic potential of targeting autophagy in the prevention and treatment of myocardial hypoxia and related heart diseases.

## Materials and Methods

### Animal Model and Procedures

Healthy male C57BL/6 mice (8–10 weeks old, weighing 18–22 g) were purchased from the Animal Center, Army Medical University (Third Military Medical University). The animals were fed with a standard diet and watered and housed under a 12 h light/dark cycle. Twenty-eight mice were allowed to acclimatize for 1 week before the experiments and were randomly divided into four groups: control, control+Baf A1 (bafilomycin A1, Farmingdale, United States), hypoxia, and hypoxia+Baf A1. The mice in the control +Baf A1 and hypoxia+Baf A1 groups were intraperitoneally injected once every other day with bafilomycin A1 (0.3 mg/kg) three times. For hypoxic exposure, mice were raised in an incubator filled with 7.5% O2 for 5 days (Dzal and Milsom, 2019). Meanwhile, the mice in control or Baf A1 group were subjected to the same procedures except to hypoxia. The mice immediately received inhalation anesthesia with 1% isoflurane (Rui Wo De, Shenzhen, China) and 7.5% O2 for echocardiography and were euthanized for collection of the myocardium samples for immunoblotting or co-immunoprecipitation analysis.

### Cardiomyocyte Cultures and Hypoxia

Neonatal Sprague–Dawley rats (1–3 days old) were purchased from the Animal Center of the Army Medical University (Third Military Medical University). Neonatal rat ventricular cardiomyocytes were isolated and incubated as previously described (Hu et al., 2010). To achieve hypoxia, the cells were cultured in the incubator filled with 94% N2, 5% CO2, and 1% O2 for 6, 9, or 12 h. The cells in the control group were incubated in a normoxic environment composed of 5% CO2 at 37°C for similar periods.

### Echocardiography Analysis

Echocardiography analysis was conducted based on our previous paper (Li et al., 2018). A mixture of isoflurane and oxygen were applied to anesthetize the mice. Criteria of cardiac function was tested by echocardiography with a Vivid 7 (GE Medical Systems, Chicago, IL, United States) instrument. The images were gathered from the view of the typical parasternal long-axis, apical four-chamber, and apical five-chamber. The GE Medical Systems software was used for data acquisition and further analysis.

### Adenovirus Infection

LC3-OE adenovirus, RIPK1-OE adenovirus, RIPK3-OE adenovirus and LC3-KD adenovirus were purchased from Genechem (Shanghai, China). Meanwhile, the ATG5-KD adenovirus and ATG5-OE adenovirus were purchased from Obio Technology (Shanghai, China). mCherry-GFP-LC3 adenoviruses were purchased from Hanbio Biotechnology (Shanghai, China). Cardiomyocytes, cultured as before (Hu et al., 2010), were transfected with adenoviruses for 48 h, and CMV-null adenoviruses were used as negative controls. Infection efficiency was determined by western blotting.

### Gene Silencing With siRNAs

siRIPK1 and siRIPK3 were purchased from Genechem (Shanghai, China). Cardiomyocytes were transfected with the targeting siRNAs or the negative control siRNAs using Lipofectamine 2000 (Invitrogen, Carlsbad, CA, United States) according to the manufacturer’s instructions. All experiments were conducted after 48 h.

### Western Blotting Assay

Left ventricular (LV) myocardium samples or cardiomyocytes were harvested in radioimmunoprecipitation assay (RIPA) buffer with protease inhibitor tablets (Beyotime, Shanghai, China) and then sonicated on ice. The lysate was then centrifuged at 14,000 rpm for 15 min at 4°C, and the supernatant was collected. Protein concentrations were assayed according to a previously published protocol using the Quick Start Bradford 1 × dye reagent (Bio-Rad, Hercules, CA, United States) (Cui et al., 2020). Proteins were separated using an SDS-PAGE gel (Bio-Rad) and then transferred to polyvinylidene fluoride membranes (Millipore, Burlington, MA, United States), where they were blocked with 5% skim milk. Then, the membranes were incubated at 4 °C overnight with the corresponding primary antibodies and horseradish peroxidase-conjugated secondary antibodies. Specific protein bands were visualized using the Western Bright Sirius chemiluminescent HRP substrate (Pierce, Waltham, MA, United States) with a ChemiDoc XRS image detector (Bio-Rad). After 1:1,000 dilution, the following antibodies were used in this experiment: rabbit polyclonal anti-LC3B (L7543, Sigma-Aldrich, St. Louis, MO, United States), anti-SQSTM1/p62 (5114, Cell Signaling Technology, Danvers, MA, United States), anti-RIPK1(17519-1-AP, Proteintech Group, Rosemont, IL, United States), anti-RIPK3 (374639, Santa Cruz Biotechnology, Dallas, TX, United States), anti-p-RIPK3 (AF7443 Affinity Biotech, Cincinnati, OH, United States), anti-p-MLKL (AF7420, Affinity Biotech), anti-GAPDH (60004, Proteintech Group), anti-ATG5 (12994, Cell Signaling Technology), anti-GST (CSB-MA000304, CusAb, Wuhan, China), anti-His-tag (CSB-MA00159, CusAb, Wuhan, China), rabbit monoclonal mouse monoclonal anti-beta-actin (ab8226, Abcam, Cambridge, United Kingdom).

### Immunoprecipitation

To determine the protein interaction between LC3 and RIPK1 or RIPK3, cardiomyocytes were lysed in RIPA buffer with a protease inhibitor tablets. The LC3B (L7543, Sigma-Aldrich) or RIPK1 (17519-1-AP, Proteintech Group) antibody was incubated with the cell lysate for 6 h at 4°C. Rabbit—derived IgG (B900610, Proteintech Group) was used as a negative control. Then, the mixture was precipitated with protein A/G-Sepharose (Santa Cruz Biotechnology) overnight at 4°C. The precipitates were washed 5 times with phosphate-buffered saline (PBS) at 4°C and separated by sodium dodecyl sulfate-polyacrylamide gel electrophoresis (SDS-PAGE) and probed using rabbit antibody by western blotting.

### GST Pull-Down Assay

GST pull-down assays were performed by the Wuhan GeneCreate Biological Engineering CO., Ltd. In brief, *lc3* gene was inserted into pGEX-6p-1 and the *ripk1* and *ripk3* genes were inserted into pET-SUMO. The three recombinant plasmids were expressed in an *Escherichia coli* expression system, which was followed by protein purification when GST-LC3, His-RIPK1, His-RIPK1(Mut1), His-RIPK1(Mut2), His-RIPK1(Mut3), His-RIPK3, His-RIPK3(Mut1), His-RIPK3(Mut2), and His-RIPK3(Mut3) was detected in the system. Mixed GST/His-RIPK(1/3/muts) and GST-LC3/His-RIPK(1/3/muts) proteins were measured and used as Input. The Pull-down assay was performed between simple GST tag proteins and His coupled RIPK1 or RIPK3 proteins performed as the negative control. GST pull-down was performed as previously described and analyzed by western blotting (Nishi et al., 2018), and detailed experimental procedure was put in Supplementary Material.

### Electron Microscopy

The procedure of this part was performed based on a previous work (Zhang et al., 2019). Cardiomyocytes were fixed in 2.5% glutaraldehyde, dehydrated, sliced with a vibratome, recut on a microtome and stained with uranyl acetate and lead citrate overnight. The sections of cardiomyocytes were visualized by transmission electron microscopy (TEM) (TECNAI 12, Philips, Amsterdam, Netherlands).

### Immunofluorescence and Confocal Microscopy

Cardiomyocytes were plated on glass coverslips, fixed with 4% paraformaldehyde for 10 min and blocked with 5% bovine serum albumin in PBS for 1 h at room temperature. Then, the cells were incubated with specific primary antibodies at 4°C overnight and were subsequently incubated with the corresponding secondary antibodies for 1 h at 37°C. The nuclei were stained for 5 min with 4′,6-diamidino-2-phenylindole (DAPI). Cells were imaged using a confocal microscope. The following primary antibodies were used in this experiment: mouse monoclonal anti-P-MLKL (AF7420, Affinity Biosciences), anti-RIPK1 (17519-1-AP, Proteintech Group), anti-RIPK1 (ab72139, Abcam), anti-RIPK3 (374639, Santa Cruz Biotechnology), anti-RIPK3 (ab62344, Abcam) and anti-LC3B (L7543, Sigma-Aldrich). The following secondary antibodies were purchased from Invitrogen: Alexa Flour 488 donkey anti-rabbit (A21206), Alexa Flour 568 donkey anti-mouse (A10037), and Alexa Flour 680 donkey anti-rabbit (A32802).

### Cell Toxicity Assays

Cytotoxicity was detected with a CytoTox-ONE C homogeneous membrane integrity assay kit (Promega, Madison, WI, United States), which is a fluorometric method used to measure the amount of LDH released into the medium from inactive cells. All experiments were performed according to the manufacturer’s instructions and repeated three times. The percentage of LDH released into the medium were used to reflect cytotoxicity.

### Statistical Analysis

The SPSS (Statistical Package for the Social Sciences) 22.0 and Image J 1.52v software were used to statistical analysis. Significant differences were determined by the unpaired Student’s *t*-test or one-way analysis of variance (ANOVA) followed by *post hoc* tests. The statistical charts were made using GraphPad. *P* < 0.05 was considered statistically significant for all comparisons.

## Results

### Necroptosis Mediates Cardiac Dysfunction Caused by Hypoxia

**FIGURE 1:**
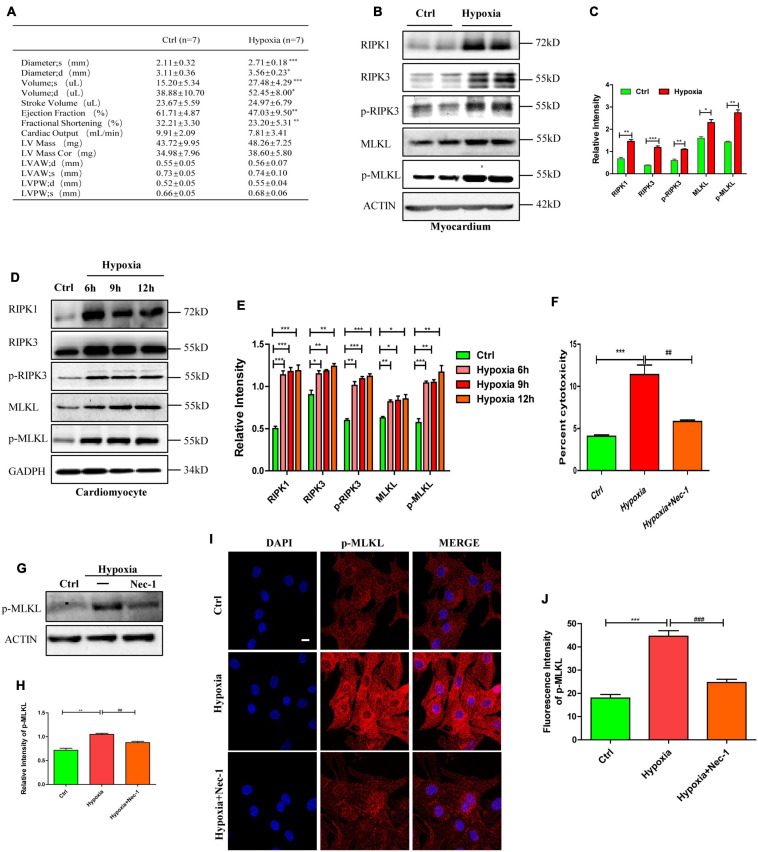
Necroptosis mediates cardiac dysfunction caused by hypoxia. **(A)** Echocardiography was performed to evaluate cardiac function after hypoxia treatment. Means ± SEM, *n* = 7. *p < 0.05, **p < 0.01, and ***p < 0.001 versus the control group. **(B,C)** Representative bands of western blotting and statistical analysis, which were performed to detect RIPK1, RIPK3, p-RIPK3, MLKL, and p-MLKL levels after hypoxia treatment in the myocardium, Means ± SEM, *n* = 5. *p < 0.05, **p < 0.01, and ***p < 0.001 versus the control group. **(D,E)** Representative immunoblotting bands and statistical analysis of RIPK1, RIPK3, p-RIPK3, MLKL, and p-MLKL in cardiomyocytes after hypoxic treatment for different durations, *n* = 5. *p < 0.05, **p < 0.01, and ***p < 0.001 versus the control group. **(F)** Lactate dehydrogenase (LDH) leakage analysis was performed to determine cell death. Mean ± SEM, *n* = 5. ***p < 0.001 versus the control group, ^##^*p* < 0.01 versus the hypoxia group. **(G,H)** Immunostaining and statistical analysis of p-MLKL from Ctrl, Hypoxia, and Hypoxia+Nec-1 groups. Mean ± SEM, *n* = 3. **p < 0.01 versus the Ctrl group, ^##^*p* < 0.01 versus the Hypoxia group. **(I,J)** Representative confocal images and statistical analysis of p-MLKL after hypoxia treatment for 9 h. Scale bar, 10 μm. *n* = 3, Mean ± SEM. ***p < 0.001 versus the control group, ^###^*p* < 0.001 versus the hypoxia group.

To investigate the effects of hypoxia on cardiac function and necroptosis *in vivo*, we exposed male C57BL/6 mice (8–10 weeks old, weighting 18–22 g) to hypoxia (7.5% O2) for 5 days. Echocardiography was performed on all mice to evaluate cardiac function *in vivo*. Decreased ejection fraction and fractional shortening (Figure 1A, *p* < 0.05) suggest that hypoxia induced cardiac dysfunction *in vivo*. To determine the effects of hypoxia *in vitro*, primary cardiomyocytes of Neonatal Sprague-Dawley rats (1–3 days old) were subjected to 1% O_2_ for 6, 9, or 12 h, respectively. Western blotting was then performed to detect the effect of hypoxia on necroptosis-related proteins *in vitro* and *in vivo*. The levels of RIPK1, RIPK3, p-RIPK3, MLKL, and p-MLKL significantly increased in the myocardium of hypoxia-treated mice (Figures 1B,C, *p* < 0.05) and in hypoxic cardiomyocytes (Figures 1D,E, *p* < 0.05), compared to the controls. In addition, cytotoxicity, measured through the lactate dehydrogenase (LDH) release assay, was significantly higher in hypoxic cardiomyocytes than in control cardiomyocytes, and cytotoxicity was reduced when the cardiomyocytes were pre-treated with Nec-1 (a necroptosis inhibitor) (Figure 1F, *p* < 0.05). The increase of p-MLKL protein level induced by hypoxia was also inhibited by Nec-1 (Figures 1G,H, *p* < 0.05). Furthermore, p-MLKL antibody was used to dye the cardiomyocytes plated on glass coverslips to measure p-MLKL level, and enhanced fluorescence intensity of p-MLKL was found in hypoxic cardiomyocytes, and it was reduced when the cardiomyocytes were pre-treated with Nec-1, indicating hypoxia augmented necroptosis (Figures 1I,J, *p* < 0.05). These data indicate that hypoxia can cause myocardial necroptosis and cardiac dysfunction simultaneously *in vitro* and *in vivo*, and inhibition of necroptosis can reduce the cytotoxicity of hypoxic cardiomyocytes. Therefore, necroptosis plays an important role in cardiac dysfunction caused by hypoxia.

### RIPK1 and RIPK3 Mediates Necroptosis in Hypoxic Cardiomyocytes

**FIGURE 2:**
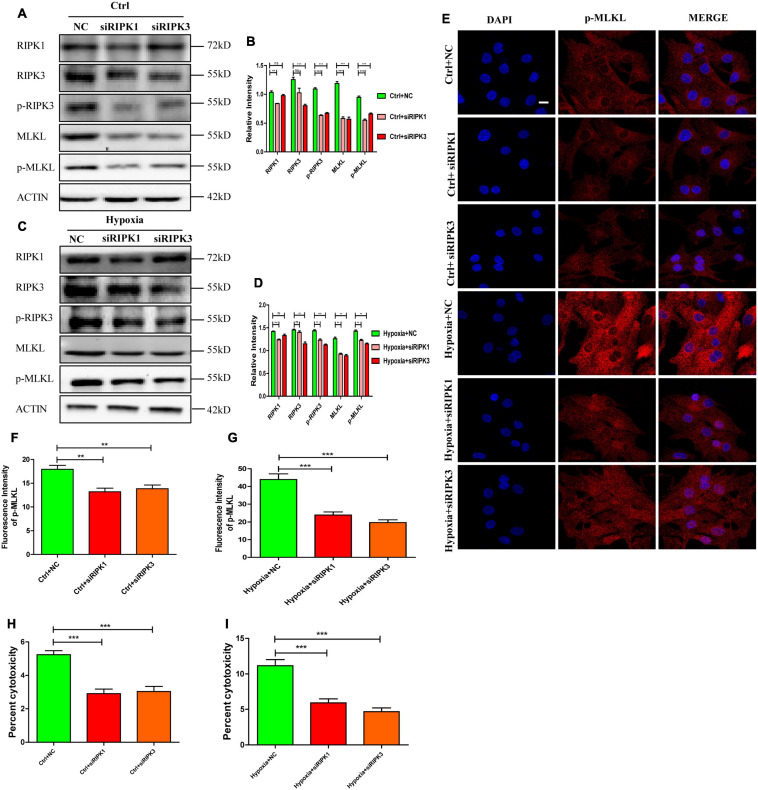
RIPK1 and RIPK3 mediates necroptosis in hypoxic cardiomyocytes. **(A–D)** siRIPK1 and siRIPK3 were applied to disrupt necroptosis both under normoxia and hypoxia. Immunostaining bands and statistical analysis of RIPK1, RIPK3, p-RIPK3, MLKL, and p-MLKL in normoxic or hypoxic cardiomyocytes. Mean ± SEM, *n* = 3. Ns means no statistical difference, **p < 0.01 and ***p < 0.001 versus the NC group. **(E–G)**. Representative confocal images and statistical analysis of p-MLKL in corresponding groups in panels **(A–D)** Scale bar, 10 μm, Mean ± SEM, *n* = 3. **p < 0.01 and ***p < 0.001 versus the NC group. **(H)** LDH leakage analysis was performed to determine cell death after addition of siRIPK1 and siRIPK3 under normoxia. Mean ± SEM, *n* = 5. ***p < 0.001 versus the NC group. **(I)** LDH leakage analysis was performed to determine cell death after addition of siRIPK1 and siRIPK3 under hypoxia. Mean ± SEM, *n* = 5. ***p < 0.001 versus the Hypoxia+NC group.

To verify the importance of RIPK1 and RIPK3 in mediating necroptosis in hypoxic cardiomyocytes, siRNA targeting RIPK1 and RIPK3 were incubated with cardiomyocytes for 48 h before hypoxia. Transfection efficiency of siRNAs was determined by western blotting (Figures 2A,B). As shown in Figures 2A,B, siRIPK1 and siRIPK3 decreased p-RIPK3 and p-MLKL levels under normoxia (*p* < 0.05). Meanwhile, the increased p-RIPK3 and p-MLKL levels caused by hypoxia were also significantly decreased by siRIPK1 and siRIPK3 (Figures 2C,D *p* < 0.05). Furthermore, fluorescent staining showed that p-MLKL was decreased by siRIPK1 and siRIPK3 under both normoxia and hypoxia (Figures 2E–G). Similarly, cytotoxicity was also decreased by siRIPK1 and siRIPK3 under hypoxia or normoxia (Figures 2H,I). The data suggest that RIPK1 and RIPK3 play important roles in regulating necroptosis in hypoxic cardiomyocytes.

### Impaired Autophagic Flux Leads to Autophagosome Accumulation and Necroptosis Under Hypoxia *in vitro* and *in vivo*

**FIGURE 3:**
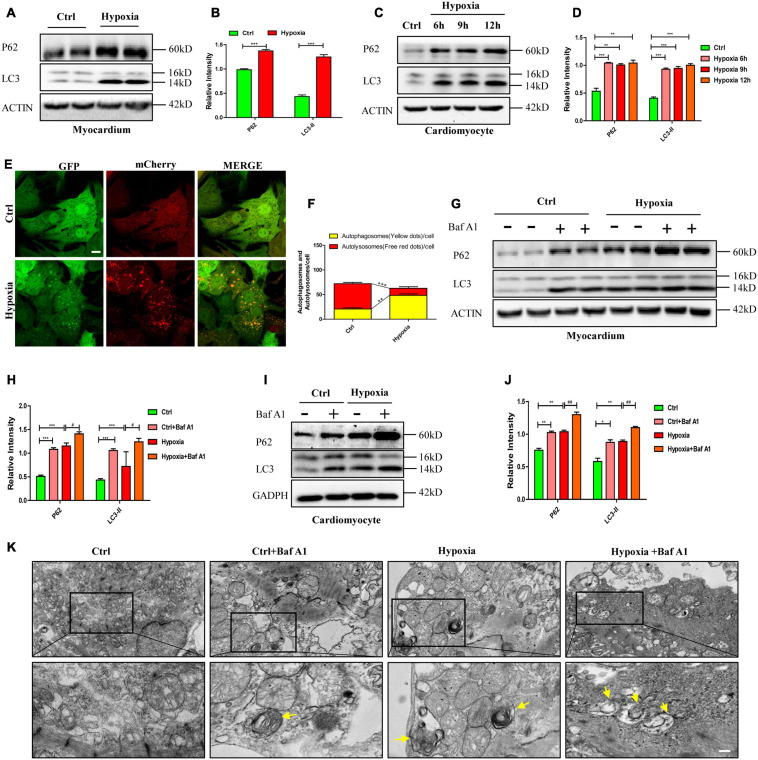
Impaired autophagic flux leads to autophagosome accumulation and necroptosis under hypoxia *in vitro* and *in vivo*. **(A,B)** Representative immunoblotting bands and statistical analysis of p62 and LC3 levels after hypoxia treatment in mouse myocardium. Mean ± SEM, *n* = 3. ***p < 0.001 versus the control group. **(C,D)** Representative immunoblotting bands and statistical analysis of p62 and LC3 levels after hypoxia treatment in cardiomyocytes. Mean ± SEM, *n* = 3. **p < 0.01, and ***p < 0.001 versus the control group. **(E,F)** Representative confocal images and statistical analysis of autophagic flux measured by mCherry-GFP-LC3 in cardiomyocytes. Scale bar, 10 μm, Mean ± SEM, *n* = 3. **p < 0.01, and ***p < 0.001. **(G,H)** Representative immunoblotting bands and statistical analysis of p62 and LC3 levels in mouse myocardium under normoxia and hypoxia with or without bafilomycin A1 (Baf A1). Mean ± SEM, *n* = 3. ***p < 0.001 and ^#^*p* < 0.05. **(I,J)** Representative immunoblotting bands and statistical analysis of p62 and LC3 levels in normoxia and hypoxia cardiomyocytes with or without Baf A1. Mean ± SEM, *n* = 3. *p < 0.05, **p < 0.01 versus the control group and ^##^*p* < 0.01 versus the hypoxia group. **(K)** Representative images of autophagosomes analyzed by transmission electron microscopy (TEM) in cardiomyocytes exposed to normoxia and hypoxia with or without Baf A1. Yellow arrow indicates the autophagosome. Scale bar, 0.5 μm, *n* = 3.

To investigate the mechanism of hypoxia-induced necroptosis in cardiomyocytes, we focused on the changes in autophagic flux, which plays an essential role in maintaining cardiac function (Liu et al., 2017). After same treatment as mentioned above, we explored the changes in the expression levels of autophagy-related proteins by western blot. As shown in Figures 3A–D, the expression levels of autophagy markers (LC3-II and p62) significantly increased in the myocardium and in cardiomyocytes after hypoxic exposure (*p* < 0.05). Furthermore, mCherry-GFP-tagged LC3 adenovirus was a typical measure to test autophagy flux. As GFP was quenched in the acidic environment of lysosome, small quantities of yellow dots would be observed under the Fluorescence microscope when autophagy flux was unobstructed. However, in present study, yellow dots were more abundant in the hypoxia group than in the control group, which had more free red dots (Figures 3E,F, *p* < 0.05). These data demonstrate that hypoxia-induced autophagic flux disruption accounts for autophagosome accumulation in cardiomyocytes.

To further determine whether impaired autophagic flux caused by hypoxia resulted in autophagosome accumulation, we applied bafilomycin A1 (Baf A1) to simulate autophagic flux impairment as Baf A1 can prevent the maturation of autophagic vacuoles by inhibiting the fusion of autophagosomes and lysosomes (Yamamoto et al., 1998). As shown in Figures 3G,H, whether under hypoxia or normoxia, myocardium treated with Baf A1 (0.3 mg/kg), had higher expressions of LC3-II and p62 than the myocardium without Baf A1 treatment (*p* < 0.05). Similarly, increased levels of LC3-II and p62 were detected in cardiomyocytes only exposed to Baf A1 or to hypoxia (Figures 3I,J, *p* > 0.05). In addition, more autophagosomes, visualized by transmission electron microscopy (TEM), were observed in the Baf A1 and hypoxia co-treated group than in the hypoxia-only group (Figure 3K). These data suggest that hypoxia and Baf A1 have similar effects on autophagy flux in cardiomyocytes.

These results indicated that autophagosome accumulation was caused by impaired autophagic flux in hypoxic cardiomyocytes and myocardium, which may drive necroptosis in hypoxic conditions.

### Autophagosome Accumulation-Induced LC3 Overexpression Provides a Spatial Platform for Necrosome Formation Through LIR Domain Mediated LC3-RIPK1 and LC3-RIPK3 Direct Interaction

As reported previously, some studies have suggested that autophagosomes were cytotoxic, but the exact mechanism was unclear (Button et al., 2017; Ogasawara et al., 2017; Dong et al., 2018). Previous studies have confirmed that the interface formed by the N-terminal and C-terminal of LC3 molecules can interact with LC3-interacting region (LIR)-containing proteins (Goold et al., 2013; Fracchiolla et al., 2016). LIR is a short linear motif of up to 13 amino acids consisting of a core sequence with a generic formula of Θ-X-X-Γ, where Θ is an aromatic amino acid (W/F/Y), Γ is a hydrophobic amino acid (L/I/V), and X could be any amino acid (Goold et al., 2013; Fracchiolla et al., 2016). Here, we determined whether RIPK1 and RIPK3 contained the LIR motif through protein sequence alignment. The results showed that RIPK1 and RIPK3 contain three identical protein sequences that from the LIR motif which is conserved in various species, including mouse, human, rat, rabbit and pig (Supplementary Figures 1A,B).

**FIGURE 4:**
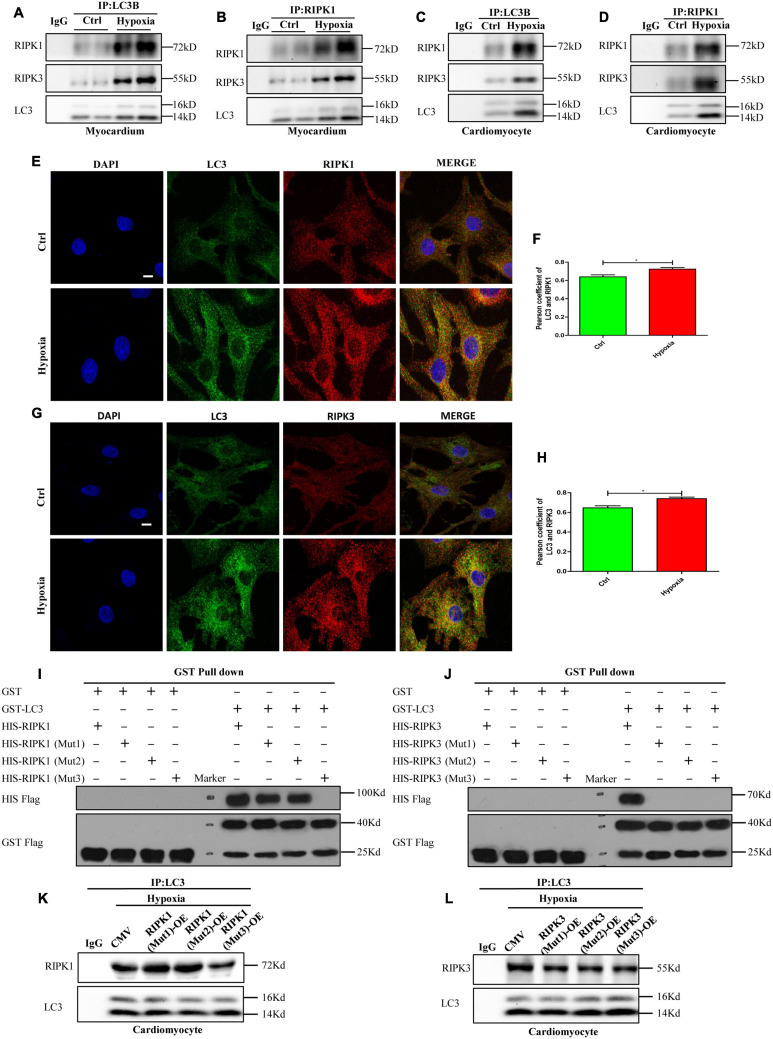
Autophagosome accumulation-induced LC3 overexpression provides a spatial platform for necrosome formation by direct interaction with RIPK1 and RIPK3. **(A,B)** The myocardium was lysed and immunoprecipitated with anti-LC3 or anti-RIPK1 antibodies followed by immunoblotting with anti-LC3, anti-RIPK1 or anti-RIPK3 antibody (*n* = 3). **(C,D)** The cardiomyocytes were lysed and immunoprecipitated with anti-LC3 or anti-RIPK1 antibodies, followed by immunoblotting with anti-LC3, anti-RIPK1 or anti-RIPK3 antibody (*n* = 3). **(E,F)** Representative confocal images and statistical analysis of Pearson coefficient of co-localizing RIPK1 (red) and LC3 (green) in cardiomyocytes. Mean ± SEM, *n* = 3. *p < 0.05 versus control group. Scale bar, 10 μm, *n* = 3. **(G,H)** Representative confocal images and statistical analysis of Pearson coefficient of co-localizing RIPK3 (red) and LC3 (green) in cardiomyocytes. Mean ± SEM. *p < 0.05 versus control group. Scale bar, 10 μm, *n* = 3. **(I,J)** The GST pull-down assay was performed with GST, GST-LC3, His-RIPK1, and His-RIPK3, before or after LIR domains were mutated. **(K,L)** The cardiomyocytes were lysed and immunoprecipitated with anti-LC3 antibodies, followed by immunoblotting with anti-LC3, anti-RIPK1 or anti-RIPK3 antibody (*n* = 3).

Therefore, to further confirm the interaction between LC3 and RIPK1/3, immunoblot and immunoprecipitation assays were performed on the hypoxia-exposed myocardium and cardiomyocytes. As shown in Figures 4A–D, and interactions between LC3, RIPK1, and RIPK3 were observed in myocardium and cardiomyocytes both under normoxia and hypoxia. Furthermore, when compared with the control group, enhanced fluorescence intensities of LC3, RIPK1, and RIPK3 were observed in cardiomyocytes treated with hypoxia, suggesting increased expressions of these proteins. Besides, LC3, RIPK1, and RIPK3 antibodies were used to dye the cardiomyocytes plated on glass coverslips to measure the co-localizations of these molecules. Colocalization of LC3-RIPK1 and LC3-RIPK3 was tested by Pearson coefficient. And Pearson coefficient value was higher than 0.6, illustrating mutual effects of LC3-RIPK1 and LC3-RIPK3 (Figures 4E–H; Bolte and Cordelières, 2006; González-Domínguez et al., 2018). To further determine whether direct interactions occur between LC3 and RIPK1/3, exogenously expressed GST-LC3, His-RIPK1, His-RIPK3, and His-RIPK1 mutations and His-RIPK3 mutations were produced and measured (Supplementary Figures 2A–E, 3A–C), and then used for glutathione S-transferase (GST) pull-down assays. Results indicate that LC3 directly interacts with RIPK1 and RIPK3 *in vitro*. Further, mutations of the RIPK1 and RIPK3 LIR domains according to Supplementary Figures 1A,B were able to alter these interactions. Specifically, W165-L168 mutation (RIPK1 Mut1) and F215-V218 mutation (RIPK1 Mut2) of RIPK1 failed to impact LC3-RIPK1 direct interaction. However, the LC3-RIPK1 interaction was vanished after F279-I282 of RIPK1 was mutated (RIPK1 Mut3). Simultaneously, no matter W115-L118 mutation (RIPK3 Mut1), F212-L215 mutation (RIPK3 Mut2), or W217-L220 mutation (RIPK3 Mut3) of RIPK3 led to the vanish of the LC3-RIPK3 interaction (Figures 4I,J). To verify the biological function of LIR domain in cardiomyocyte, RIPK1-OE (Mut1)/(Mut2)/(Mut3) and RIPK3-OE (Mut1)/(Mut2)/(Mut3) adenovirus were constructed. Infection efficiency of these adenovirus was determined by western blotting after incubation for 48 h. The results showed that all overexpression adenoviruses were effective (Supplementary Figures 4A–L, *p* < 0.05). The increased content of RIPK1 caused by RIPK1-OE (Mut1) and RIPK1-OE (Mut2) adenovirus under normoxic conditions became more obvious under hypoxia (p < 0.05). However, the content of RIPK1 increased by RIPK1-OE (Mut3) adenovirus under normoxic conditions was not augmented by hypoxia (*p* > 0.05). Meanwhile, the content of RIPK3 increased by RIPK3-OE (Mut1), RIPK3-OE (Mut2) and RIPK3-OE (Mut3) adenovirus under normoxic conditions was not augmented by hypoxia (Supplementary Figures 4A–L, *p* > 0.05). When compared with hypoxia + CMV group, the interaction between LC3 and RIPK1 was decreased in hypoxia+RIPK1-OE (Mut3) group, but not in hypoxia+RIPK1-OE (Mut1) and hypoxia+RIPK1-OE (Mut2) groups (Figure 4K). Further, either RIPK3-OE (Mut1), RIPK3-OE (Mut2) or RIPK3-OE (Mut3) adenovirus decreased the interaction between LC3 and RIPK3 in hypoxia (Figure 4L). These results indicated that RIPK1 contained one LIR domain like P62 and NDP52 molecules. While RIPK3 contained more than one LIR domains like NBR1 and STING molecules, specifically three LIR domains (Birgisdottir et al., 2013; Liu et al., 2019). And LIR domain plays an essential role both in the interaction of LC3-RIPK1 and LC3-RIPK3.

**FIGURE 5:**
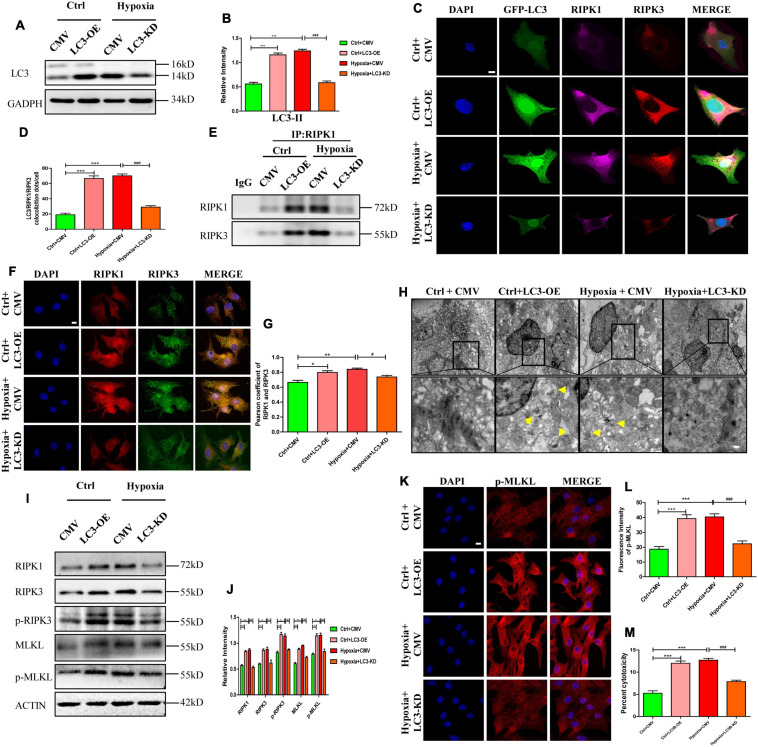
RIPK1 and RIPK3 augmentation induced by autophagosome accumulation facilitates necroptosis under hypoxic conditions. **(A,B)** Infection efficiency of LC3 overexpression (OE), LC3 knockdown (KD), and MCMV-null adenoviruses were determined by western blotting. Mean ± SEM, *n* = 3. ***p < 0.001 versus the control+CMV group. ^###^*p* < 0.001 versus the hypoxia+CMV group. **(C,D)** Co-localization and statistical analysis of LC3, RIPK1 and RIPK3 in groups from panel **(A)**. Mean ± SEM, ***p < 0.001 versus the Ctrl+CMV group, ^###^*p* < 0.001 versus the Hypoxia+CMV group Scale bar, 10 μm, *n* = 3. **(E)** After regulating autophagy with LC3 overexpression or knockdown adenovirus under normoxia or hypoxia, protein samples were collected from cardiomyocytes and immunoprecipitated with anti-RIPK1 antibodies, followed by immunoblotting with anti-RIPK1 or anti-RIPK3 antibody (*n* = 5). **(F,G)** Co-localization and statistical analysis of RIPK1 and RIPK3 in groups from panel **(A)**. *p < 0.05 and **p < 0.001 versus the Ctrl+CMV group, ^#^*p* < 0.05 versus the Hypoxia+CMV group. Scale bar, 10 μm, *n* = 3. **(H)** Representative images of autophagosomes in corresponding groups in panel **(D)**. Yellow arrow points to an autophagosome. Scale bar, 0.5 μm, *n* = 3. **(I,J)** Western blotting and statistical analysis of RIPK1, RIPK3, p-RIPK3, MLKL, and p-MLKL from Ctrl+CMV, Ctrl+LC3-OE, Hypoxia+CMV, and Hypoxia+LC3-KD groups (*n* = 3). Mean ± SEM. *p < 0.05, **p < 0.01, ***p < 0.001 versus the Ctrl+CMV group, ^#^*p* < 0.05 and ^##^*p* < 0.01 versus the Hypoxia+CMV group. **(K,L)** Representative confocal images and statistical analysis of p-MLKL in corresponding groups in panel **(A)**. Mean ± SEM. ***p < 0.001 versus the Ctrl+CMV group, ^###^*p* < 0.001 versus the Hypoxia+CMV group. Scale bar, 10 μm, *n* = 3. **(M)** Lactate dehydrogenase (LDH) leakage analysis was performed to determine cell death. Mean ± SEM, *n* = 5. ***p < 0.001 versus the Ctrl+CMV group, ^###^*p* < 0.001 versus the Hypoxia+CMV group.

To further identify the effects of LC3 on necrosome formation, cells were randomly divided into four groups: control+CMV, control+LC3-OE, hypoxia+CMV, and hypoxia+LC3-KD. Infection efficiency of adenovirus was determined by western blotting after incubation for 48 h. We found that the expression of LC3 was increased by LC3-OE adenovirus and was decreased by the LC3-KD adenovirus (Figures 5A,B). The co-localization of LC3-RIPK1-RIPK3 was detected by the immunofluorescence assay, the result showed that the trimer, consisted of LC3, RIPK1, and RIPK3, was increased in the LC3-OE and hypoxia group compared with the ctrl group, and it decreased in the LC3-KD group compared with hypoxia group (Figures 5C,D). The number of necrosome, consisting of RIPK1-RIPK, significantly increased in cardiomyocytes transfected with the LC3-OE adenovirus compared with corresponding CMV-null group (Figures 5E–G). In line with this, the necrosome significantly reduced by the LC3-KD adenovirus (Figures 5E–G). These results indicate that LC3 stimulates necrosome complex formation by providing a spatial platform for the aggregation of RIPK1 and RIPK3.

### RIPK1 and RIPK3 Augmentation Induced by Autophagosome Accumulation Facilitates Necroptosis Under Hypoxia

To study the effects of autophagic flux on necroptosis, necroptosis was assayed after modulating autophagic flux using LC3-related adenovirus. As shown in Figures 5A,B, after identical treatment as previous paragraph, LC3 expression in cardiomyocytes were significantly affected by LC3-OE or LC3-KD adenovirus (*p* < 0.05). Similarly, increased autophagosome levels were observed in cardiomyocytes exposed to hypoxia or to LC3-OE adenovirus (Figures 5A,B,H, *p* < 0.05). However, after pre-treatment with LC3-KD adenovirus, the hypoxia-induced increased autophagosome abundance were significantly reduced (Figures 5A,B,H, *p* < 0.05). Meanwhile, the expression levels of RIPK1 and RIPK3 in cardiomyocytes increased after treatment with LC3-OE adenovirus and exposure to hypoxia, and were markedly reduced by the LC3-KD adenovirus under hypoxic conditions (Figures 5I,J, *p* < 0.05). These results suggest that accumulated autophagosome stimulates necrosome formation, while decreased autophagosome inhibits necrosome formation. In addition, the levels of p-RIPK3 and p-MLKL were augmented by the accumulated autophagosome induced by LC3-OE adenovirus or hypoxia, and were reduced by LC3-KD adenovirus, which restored the accumulated autophagosome to a certain degree (Figures 5I,J, *p* < 0.05). Moreover, fluorescent staining of p-MLKL showed similar alterations to p-MLKL levels (Figures 5K,L). Cytotoxicity, as assessed by LDH release, increased in the LC3-OE adenovirus group but significantly decreased in the LC3-KD adenovirus group under hypoxic conditions (Figure 5M, *p* < 0.05). These data showed that accumulated autophagosome induced by LC3 overexpression or hypoxia promoted necroptosis, and decreased autophagosome induced by LC3 knockdown inhibited necroptosis, which was indicated by the LC3-associated decrease of RIPK1 and RIPK3.

**FIGURE 6:**
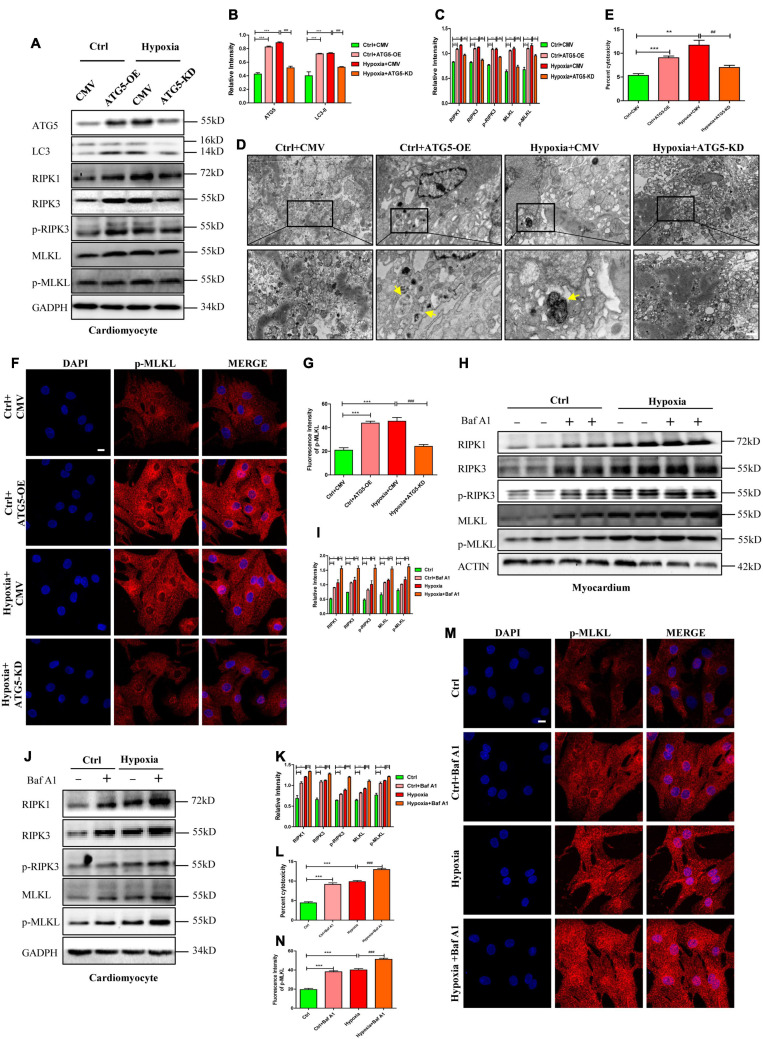
RIPK1 and RIPK3 augmentation induced by autophagic flux impairment facilitates necroptosis under hypoxia. **(A–C)** ATG5 overexpression and ATG5 knockdown adenovirus were used to regulate autophagy under normoxia or hypoxia, respectively. After that, western blot and statistical analysis was performed to detect the levels of ATG5, LC3, RIPK1, RIPK3, p-RIPK3, MLKL, and p-MLKL. Mean ± SEM, *n* = 3. **p < 0.01 and ***p < 0.001 versus the Ctrl+CMV group, ^#^*p* < 0.05, ^##^*p* < 0.01 and ^###^*p* < 0.001 versus the Hypoxia+CMV group. **(D)** Representative images of autophagosome in corresponding groups in panel **(A)**. Yellow arrow indicates an autophagosome. Scale bar, 0.5 μm, *n* = 3. **(E)** LDH leakage analysis was performed to determine cell death. Mean ± SEM, *n* = 5. **p < 0.01 and ***p < 0.001 versus the Ctrl+CMV group, ^##^*p* < 0.01 versus the Hypoxia+CMV group. **(F,G)**. Representative confocal images and statistical analysis of p-MLKL in corresponding groups in panel **(A)**. Mean ± SEM. ***p < 0.001 versus the Ctrl+CMV group, ^###^*p* < 0.001 versus the Hypoxia+CMV group. Scale bar, 10 μm, *n* = 3. **(H–K)** Bafilomycin A1 (Baf A1) was used to adjust autophagic flux under normoxia and hypoxia both *in vitro* and *in vivo*. Western blot and statistical analysis were then performed to determine the levels of RIPK1, RIPK3, p-RIPK3, MLKL, and p-MLKL in cardiomyocytes and myocardium, respectively. Mean ± SEM, *n* = 3. *p < 0.05, **p < 0.01 and ***p < 0.001versus the control group, ^#^*p* < 0.05, ^##^*p* < 0.01 and ^###^*p* < 0.001 versus the hypoxia group. **(L)** LDH leakage analysis was performed to determine cell death of corresponding groups in panel **(E)**. Mean ± SEM, *n* = 5. ***p < 0.001 versus the control group, ^###^*p* < 0.001 versus the hypoxia group. **(M,N)** Representative confocal images and statistical analysis of p-MLKL in corresponding groups in panel **(G)**. Mean ± SEM. ***p < 0.001 versus the Ctrl group, ^###^*p* < 0.001 versus the Hypoxia group. Scale bar, 10 μm, *n* = 3.

To further confirm the effects of autophagosome accumulation on necroptosis, ATG5 expression was manipulated through overexpression or knockdown before testing necroptosis. In detail, cells were randomly divided into four groups: control+CMV, control+ATG5-OE, hypoxia+CMV, and hypoxia+ATG5-KD. Infection efficiency of adenovirus was determined by western blotting after incubation for 48 h. As shown in Figures 6A–D, ATG5-OE adenovirus promoted autophagosome generation, as shown by the increased LC3-II and autophagosome, while ATG5-KD alleviated autophagosome accumulations under hypoxic conditions (*p* < 0.05). ATG5-OE also resulted in increased expression levels of RIPK1 and RIPK3, while ATG5-KD inhibited RIPK1 and RIPK3 expression. Moreover, ATG5-OE resulted in increased levels of p-RIPK3, MLKL and p-MLKL, and enhanced cytotoxicity in cardiomyocytes, while ATG5-KD lowered levels of p-RIPK3, MLKL and p-MLKL and lower cytotoxicity in cardiomyocytes under hypoxia (Figures 6A–C,E–G, *p* < 0.05). These results further suggested that autophagosome accumulations induced by ATG5 overexpression or hypoxia were able to stimulate necroptosis, and decreased autophagosome mediated by ATG5 knockdown inhibited necroptosis, which was reflected in the LC3-associated decrease in RIPK1 and RIPK3 levels. In addition, these increased indexes of necroptosis proteins, p-MLKL fluorescence intensity and cytotoxicity decreased significantly after the addition of LC3-KD adenovirus in ATG5-OE group (Supplementary Figures 5A–F). And these decreased indexes of necroptosis proteins, p-MLKL fluorescence intensity and cytotoxicity in hypoxia+ATG5-KD group increased significantly after the addition of LC3-OE adenovirus (Supplementary Figures 5G–L). These results suggest that the occurrence of cardiomyocyte necroptosis under hypoxia is closely related to the expression level of LC3 molecules.

Because Baf A1 could induce autophagosome accumulation, it was then applied to investigate the role of autophagosome accumulation on necroptosis in cardiomyocytes. Noticeably, the expression levels of RIPK1 and RIPK3 were upregulated by Baf A1 or hypoxia treatment in cardiomyocytes and the myocardium, and RIPK1 and RIPK3 increased in the Baf A1+hypoxia group when compared to the hypoxia group (Figures 6H–K). Additionally, the levels of p-RIPK3 and p-MLKL, the fluorescence intensity of p-MLKL, and cytotoxicity were all augmented in Baf A1- or hypoxia-treated groups both *in vivo* and *in vitro*, and even more obviously increased in the Baf A1+hypoxia group (Figures 6H–N). Together, these data suggest that autophagosome accumulation contributes to RIPK1 and RIPK3 augmentation, and consequently results in the formation of the necrosome, which leads to necroptosis.

## Discussion

**FIGURE 7:**
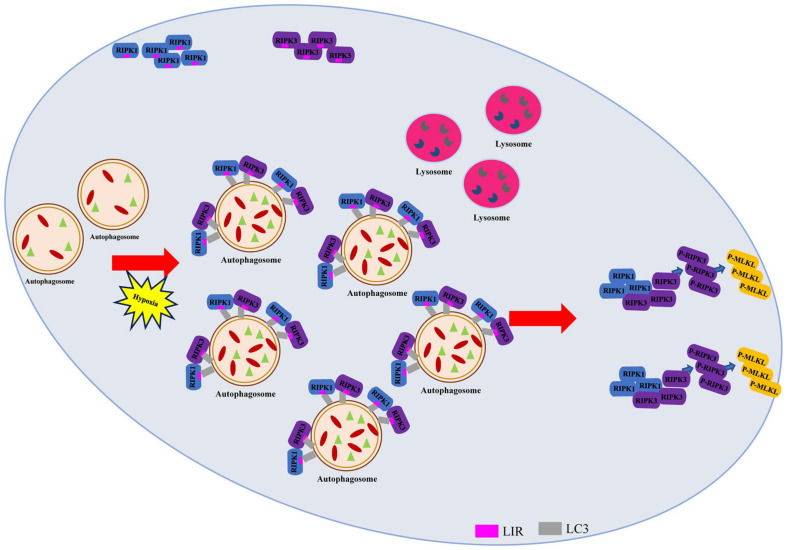
Autophagy-related LC3 accumulation stimulates necroptosis through direct interaction of LC3 with RIPK1 and RIPK3 in hypoxic cardiomyocytes. Schematic diagram on the proposed process where impaired autophagic flux contributes to necroptosis through interaction between LC3, RIPK1, and RIPK3 in hypoxic conditions.

The specific mechanisms underlying hypoxia-induced autophagic flux impairment and necroptosis activation remain unclear. In this study, we mainly analyzed the effects of autophagosome accumulation on necroptosis in hypoxic cardiomyocytes. The salient findings presented here revealed that direct interactions existed between LC3-RIPK1 and LC3-RIPK3 through LIR domain. We further discovered that these interactions led to necrosome formation, resulting in necroptosis under hypoxic conditions, during which autophagic flux was impaired and failed to lower the RIPK1 and RIPK3 levels (Figure 7). Conversely, alleviating the hypoxia-induced autophagosome accumulation with LC3-KD or ATG5-KD adenovirus resulted in necrosome clearance and necroptosis inhibition. Collectively, these findings provide a novel insight into the roles of LC3 in regulating cardiomyocyte necroptosis and indicate its therapeutic potential in the prevention and treatment of hypoxic myocardial injury and other hypoxia-related diseases.

In this study, autophagic flux was disrupted in hypoxic cardiomyocytes. In detail, increased expression levels of LC3-II and p62, and autophagosome accumulation were observed in hypoxic cardiomyocytes and myocardium. Consistent with these results, a previous report has demonstrated that insufficient oxygen impaired autophagic flux through the ROS/HIF-1α/BNIP3/NIX signaling pathway (Li et al., 2015); a study by our group also showed that autophagic flux was disrupted in hypoxic cardiomyocytes (Cui et al., 2020). In addition, results of present study revealed that hypoxia stimulated necrosome formation and necroptosis activation as reflected by the enhanced fluorescence intensity of p-MLKL and increased levels of RIPK1, RIPK3, p-RIPK3, and p-MLKL. In line with our results, previous studies have also demonstrated that necroptosis is activated in hypoxic cardiomyocytes (Zhang et al., 2016). These observations led us to further investigate the exact association between these two essential biological processes in hypoxic cardiomyocytes.

Our data here suggest that autophagosome accumulation influences necroptosis in hypoxic cardiomyocytes. We have shown that autophagosome accumulation in hypoxic cardiomyocytes contributed to necrosome formation, indicated by augmented levels of RIPK1 and RIPK3, and resulted in the activation of necroptosis. We further confirmed the phenomenon by simulating autophagosome accumulation using Baf A1 *in vitro* and *in vivo*. In line with this, Liu et al. (2018) have reported that lysosomal dysfunction, one of the causes of autophagic flux impairment and autophagosome accumulation, sensitized cells to necroptosis by promoting RIPK1 and RIPK3 accumulation in a spinal cord injury model. Furthermore, increased levels of p62, another indicator of autophagic flux disruption and autophagosome accumulation, contributed to necrosome assembly by recruiting RIPK1, which resulted in necroptosis in *Map3k7*-deleted mouse prostate cells (Goodall et al., 2016). Although these published works supported our results, no further investigations about the effects of unobstructed autophagic flux on necroptosis were conducted. In our study, we alleviated the hypoxia-induced autophagosome accumulation using LC3 or ATG5 knockdown adenoviruses and then assayed necroptosis. Our results revealed decreased necrosome assembly and inhibited necroptosis in hypoxic cardiomyocytes when autophagosome accumulation was declined after LC3 or ATG5 knockdown. Our previous work has proved that 3-MA(3-methyladenine) can inhibit autophagy in hypoxic cardiomyocytes (Cui et al., 2020). Further, Liu et al. (2016) found that the addition of 3-MA during ischemia/reperfusion can effectively reduce necroptosis. These results suggest that it is feasible to reduce autophagosome accumulation to alleviate hypoxia induced necroptosis with chemical intervention. A protective function of smoothed autophagy flow has been previously demonstrated in cardiomyocytes, but direct evidence is yet to be found (Wang et al., 2018). Taken together, the modulatory effects of autophagy on necroptosis have been demonstrated in this study, but the underlying mechanisms still have to be determined.

Until now, LC3 has been identified as an autophagosome marker, and few studies have focused on the specific function of LC3. Phosphatidylethanolamine (PE) is required during the conversion of LC3-I to LC3-II, and PE is a key mediator of protein folding. Decreased PE activates endoplasmic reticulum stress (ERS) and cell injury (Patel and Witt, 2017). Therefore, LC3-II might be a crucial contributor of cell injury. More direct evidence provided by Mizumura et al. (2018) showed that dynamic interactions of the autophagy protein LC3B with caveolin-1 (Cav-1) and Fas regulated cigarette smoke-induced lung epithelial cell apoptosis. In our research, we used adenovirus including ATG5-OE, ATG5-KD, LC3-OE, LC3-KD, ATG5-OE/LC3-KD, and ATG5-KD/LC3-OE to treat cardiomyocytes, and the results showed that the change of protein content of LC3 molecule was closely related to the occurrence of necroptosis. These results confirmed that LC3 molecule was a functional molecule, which was consistent with the above research (Mizumura et al., 2018; Liu et al., 2019). Furthermore, studies on the structure of LC3 revealed that the interface formed by the N-terminal and C-terminal of LC3 molecules can interact with the LIR-containing proteins (Kalvari et al., 2014). LIR is a short linear motif of up to 13 amino acids whose core sequence conforms to the generic formula Θ-X-X-Γ—where Θ is an aromatic amino acid (W/F/Y), Γ is a hydrophobic amino acid (L/I/V), and X can be any amino acid (Liu et al., 2017). RIPK1 and RIPK3 are critical components of the necrosome and affect cell death, but whether they contain the LIR domain remains unclear (Cho et al., 2009). The results showed that RIPK1 and RIPK3 contained LIR domains, and LIR domains play an indispensable role in the interaction between LC3-RIPK1 and LC3-RIPK1. As for RIPK1 containing only one LIR domain and RIPK3 containing three LIR domains, we believe that this may be related to the spatial conformation of the molecule (Kronlage et al., 2019). It is possible that only one LIR domain of RIPK1 is exposed to the surface conceived in molecular space, so there is only one effective domain that interacts with LC3 molecules. On the other hand, all the LIR domains of RIPK3 molecules are exposed to the conformational surface of molecular space so that they can interact with LC3 molecules. These speculations need more evidence to confirm. The interactions between LC3, RIPK1 and RIPK3 were significantly enhanced in hypoxic cardiomyocytes and myocardium, which explains why hypoxia-induced autophagosome accumulation accelerated necrosome formation and eventually led to necroptosis.

In conclusion, our current work provides a new insight into the detailed mechanism for necrosome assembly and necroptosis induced by autophagosome accumulation in hypoxic cardiomyocytes and myocardium. In brief, we discovered that LIR domains-mediated LC3-RIPK1 and LC3-RIPK3 interactions led to necrosome formation, resulting in necroptosis under hypoxia, under which autophagic flux was impaired and was failed to lower the RIPK1 and RIPK3 levels. And the mechanism explains why undisrupted autophagic flux helps maintain cell homeostasis. Our results also imply that LC3 is an important mediator between autophagy and necroptosis. Even through, limitations are existed in this study, such as the mechanisms of degrading RIPK1 and RIPK3 when autophagy flux is unobstructed require further efforts. Based on our results, a novel therapeutic scheme targeting LC3 may help in the intervention of hypoxia-induced cardiac dysfunction or other hypoxia-related diseases.

## Data Availability Statement

The original contributions presented in the study are included in the article/Supplementary Material, further inquiries can be directed to the corresponding author/s.

## Ethics Statement

The animal study was reviewed and approved by the Animal Experiment Ethics Committee of the Army Medical University.

## Author Contributions

YH designed and conducted the whole experiment, collected and interpreted the data, and drafted the manuscript. YF, LC, and LY participated in the design and coordination of the experimental work. JZ, XJ, and XZ took part in the experimental work and the data analysis. QZ, YL, and J-ZJ taught the experimental methods. D-XZ and Y-SH were responsible for the study design, data analysis and interpretation, and manuscript modification. All authors contributed to the article and approved the submitted version.

## Conflict of Interest

The authors declare that the research was conducted in the absence of any commercial or financial relationships that could be construed as a potential conflict of interest.

## Supplementary Material

Supplementary Figure 1 Protein sequence alignment of RIPK1 and RIPK3 with LIR motif. **(A)** Three identical protein sequences forming LIR motif, marked in red, in the protein sequences of mouse, human, rat, pig, and rabbit RIPK1. ^∗^ Represents relatively conserved protein sites in different species. **(B)** Three identical protein sequences forming LIR motif, marked in red, in the protein sequences of mouse, human, rat, and pig RIPK3. ^∗^ Represents relatively conserved protein sites in different species.

Supplementary Figure 2 **(A)** GST-LC3, His-RIPK1, and His-RIPK3 were identified through Coomassie brilliant blue staining. Red arrows point to the three proteins. **(B)** Three mutated RIPK1 protein according to Supplementary Figure 1A were constructed, and identified through Coomassie brilliant blue staining. Red arrows point to the three proteins. **(C)** Three mutated RIPK3 protein according to Supplementary Figure 1B were constructed, and identified through Coomassie brilliant blue staining. Red arrows point to the three proteins. **(D)** Immunostaining of GST and GST-LC3. Red arrows point to the protein. **(E)** Immunostaining of His, His-RIPK1, and His-RIPK3. Red arrows point to the protein.

Supplementary Figure 3 **(A)** Immunostaining of His and His-RIPK1(Mut2). Red arrows point to the protein. **(B)** Immunostaining of His, His-RIPK1(Mut1), and His-RIPK1(Mut3). Red arrows point to the protein. **(C)** Immunostaining of His, His-RIPK3(Mut1), His-RIPK3(Mut2), and His-RIPK3(Mut3). Red arrows point to the protein. **(D,E)** Input of Figure4i and Figure4j, respectively. **(F,G)** Input of Figure4k and Figure4l, respectively.

Supplementary Figure 4 **(A,B)** Western blotting and statistical analysis of RIPK1 from Ctrl+CMV, Ctrl+RIPK1-OE(Mut1) and Hypoxia+RIPK1-OE(Mut1). Mean ± SEM, *n* = 3. ^∗∗∗^*p* < 0.001 versus the Ctrl+CMV group, ^##^*p* < 0.01 versus the Ctrl+RIPK1-OE(Mut1) group. **(C,D)** Western blotting and statistical analysis of RIPK1 from Ctrl+CMV, Ctrl+RIPK1-OE(Mut2) and Hypoxia+RIPK1-OE(Mut2). Mean ± SEM, *n* = 3. ^∗∗∗^*p* < 0.001 versus the Ctrl+CMV group, ^#^*p* < 0.05 versus the Ctrl+RIPK1-OE(Mut2) group. **(E,F)** Western blotting and statistical analysis of RIPK1 from Ctrl+CMV, Ctrl+RIPK1-OE(Mut3) and Hypoxia+RIPK1-OE(Mut3). Mean ± SEM, *n* = 3. ^∗∗^*p* < 0.01 versus the Ctrl+CMV group, ns means no statistical difference versus the Ctrl+RIPK1-OE(Mut3) group. **(G,H)** Western blotting and statistical analysis of RIPK3 from Ctrl+CMV, Ctrl+RIPK3-OE(Mut1), and Hypoxia+RIPK3-OE(Mut1). Mean ± SEM, *n* = 3. ^∗∗^*p* < 0.01 versus the Ctrl+CMV group, ns means no statistical difference versus the Ctrl+RIPK3-OE(Mut1) group. **(I,J)** Western blotting and statistical analysis of RIPK3 from Ctrl+CMV, Ctrl+RIPK3-OE(Mut2) and Hypoxia+RIPK3-OE(Mut2). Mean ± SEM, *n* = 3. ^∗∗∗^*p* < 0.001 versus the Ctrl+CMV group, ns means no statistical difference versus the Ctrl+RIPK3-OE(Mut2) group. **(K,L)** Western blotting and statistical analysis of RIPK3 from Ctrl+CMV, Ctrl+RIPK3-OE(Mut3) and Hypoxia+RIPK3-OE(Mut3). Mean ± SEM, *n* = 3. ^∗∗^*p* < 0.01 versus the Ctrl+CMV group, ns means no statistical difference versus the Ctrl+RIPK3-OE(Mut3) group.

Supplementary Figure 5 **(A–C)** Western blotting and statistical analysis of ATG5, LC3, RIPK1, RIPK3, p-RIPK3, MLKL and p-MLKL from Ctrl+CMV, Ctrl+ATG5-OE, and Ctrl+ATG5-OE groups. Mean ± SEM, *n* = 3. ^∗^*p* < 0.05, ^∗∗^*p* < 0.01, ^∗∗∗^*p* < 0.001 versus the Ctrl+CMV group, ^#^*p* < 0.05 and ^##^*p* < 0.01 versus the Ctrl+ATG5-OE group. **(D)** LDH leakage analysis was performed to determine cell death of corresponding groups in panel **(A)**. Mean ± SEM, *n* = 3. ^∗∗∗^*p* < 0.001 versus the Ctrl+CMV group, ^###^*p* < 0.001 versus the Ctrl+ATG5-OE group. **(E,F)** Representative confocal images and statistical analysis of p-MLKL in corresponding groups in panel **(A)** Scale bar, 10 μm, Mean ± SEM, *n* = 3. ^∗∗∗^*p* < 0.001 versus the Ctrl+CMV group, ^###^*p* < 0.001 versus the Ctrl+ATG5-OE group. **(G–I)** Western blotting and statistical analysis of ATG5, LC3, RIPK1, RIPK3, p-RIPK3, MLKL, and p-MLKL from Hypoxia+CMV, Hypoxia +ATG5-KD, and Hypoxia +ATG5-KD+LC3-OE groups. Mean ± SEM, *n* = 3. Ns means no statistical difference, ^∗∗^*p* < 0.01, ^∗∗∗^*p* < 0.001 versus the Hypoxia+CMV group, ^##^*p* < 0.01 and ^###^*p* < 0.001 versus the Hypoxia+ ATG5-KD group. **(J)** LDH leakage analysis was performed to determine cell death of corresponding groups in panel **(G)**. Mean ± SEM, *n* = 5. ^∗∗^*p* < 0.01 versus the Hypoxia+CMV group, ^##^*p* < 0.01 versus the Hypoxia +ATG5-KD group. **(K,L)** Representative confocal images and statistical analysis of p-MLKL in corresponding groups in panel **(G)** Scale bar, 10 μm, Mean ± SEM, *n* = 3. ^∗∗∗^*p* < 0.001 versus the Hypoxia+CMV group, ^###^*p* < 0.001 versus the Hypoxia +ATG5-KD group.

Supplementary Figure 6 **(A,B)** The PI staining and statistical analysis from Ctrl, Hypoxia, and Hypoxia+Nec-1 groups. Mean ± SEM, *n* = 3. ^∗∗∗^*p* < 0.001 versus the Ctrl group and ^##^*p* < 0.01 versus the Hypoxia group.

## Abbreviations

KD: knockdown
LC3: microtubule-associated protein 1A/1B-light chain 3
LDH: lactate dehydrogenase
LIR: LC3-interacting region
MLKL: mixed lineage kinase domain-like pseudokinase
OE: overexpression
RIPK: Receptor-interacting protein kinase.
