# Supplementary figures and images for: Autophagy-Related LC3 Accumulation Interacted Directly With LIR Containing RIPK1 and RIPK3, Stimulating Necroptosis in Hypoxic Cardiomyocytes
Source: Front Cell Dev Biol. 2021 Jul 23;9:679637. doi: 10.3389/fcell.2021.679637 (PMC8344065; doi:10.3389/fcell.2021.679637)

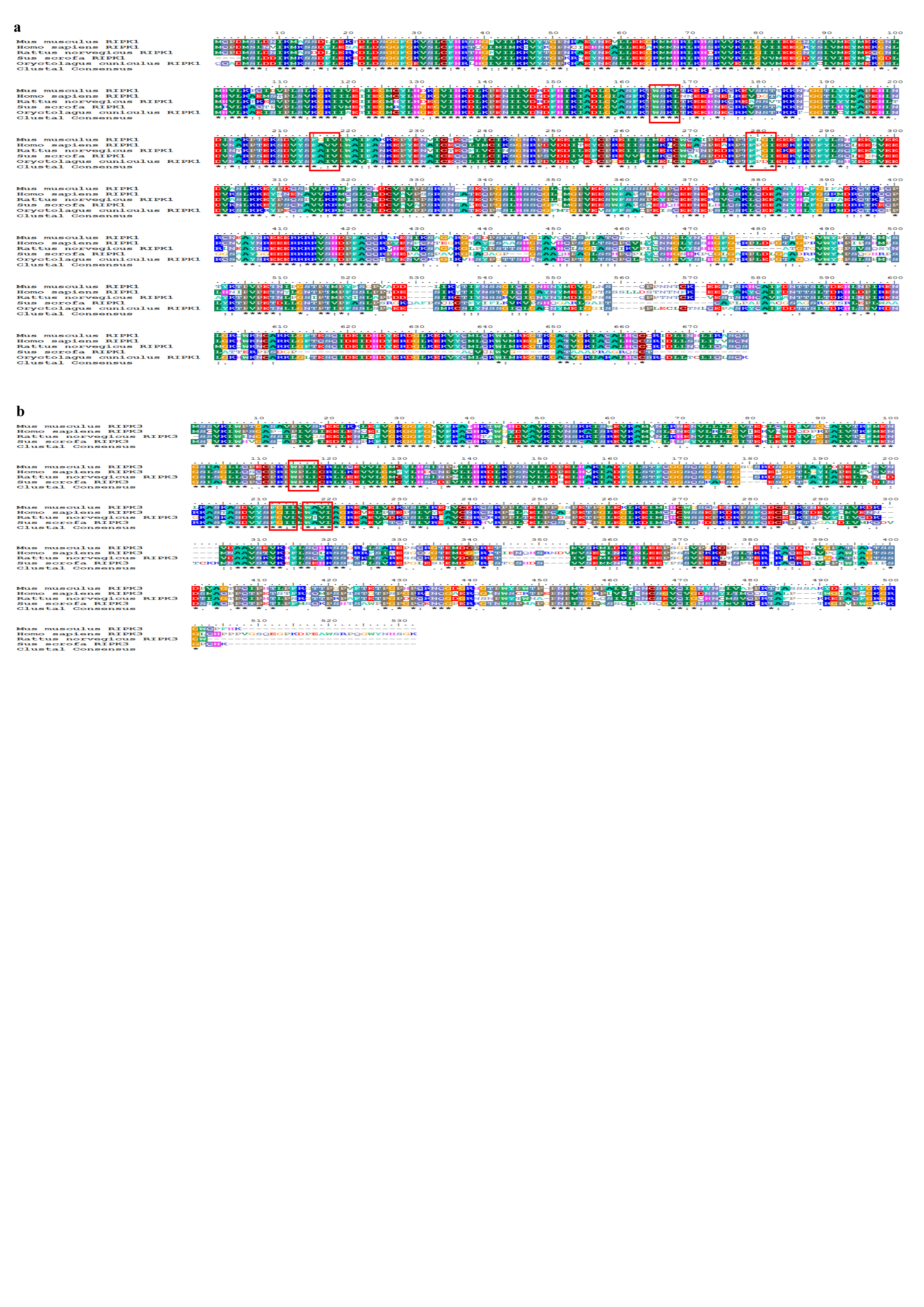

Supplement: Supplementary Figure 1 — Protein sequence alignment of RIPK1 and RIPK3 with LIR motif. (A) Three identical protein sequences forming LIR motif, marked in red, in the protein sequences of mouse, human, rat, pig, and rabbit RIPK1. ∗ Represents relatively conserved protein sites in different species. (B) Three identical protein sequences forming LIR motif, marked in red, in the protein sequences of mouse, human, rat, and pig RIPK3. ∗ Represents relatively conserved protein sites in different species. [file Image_1.TIF]

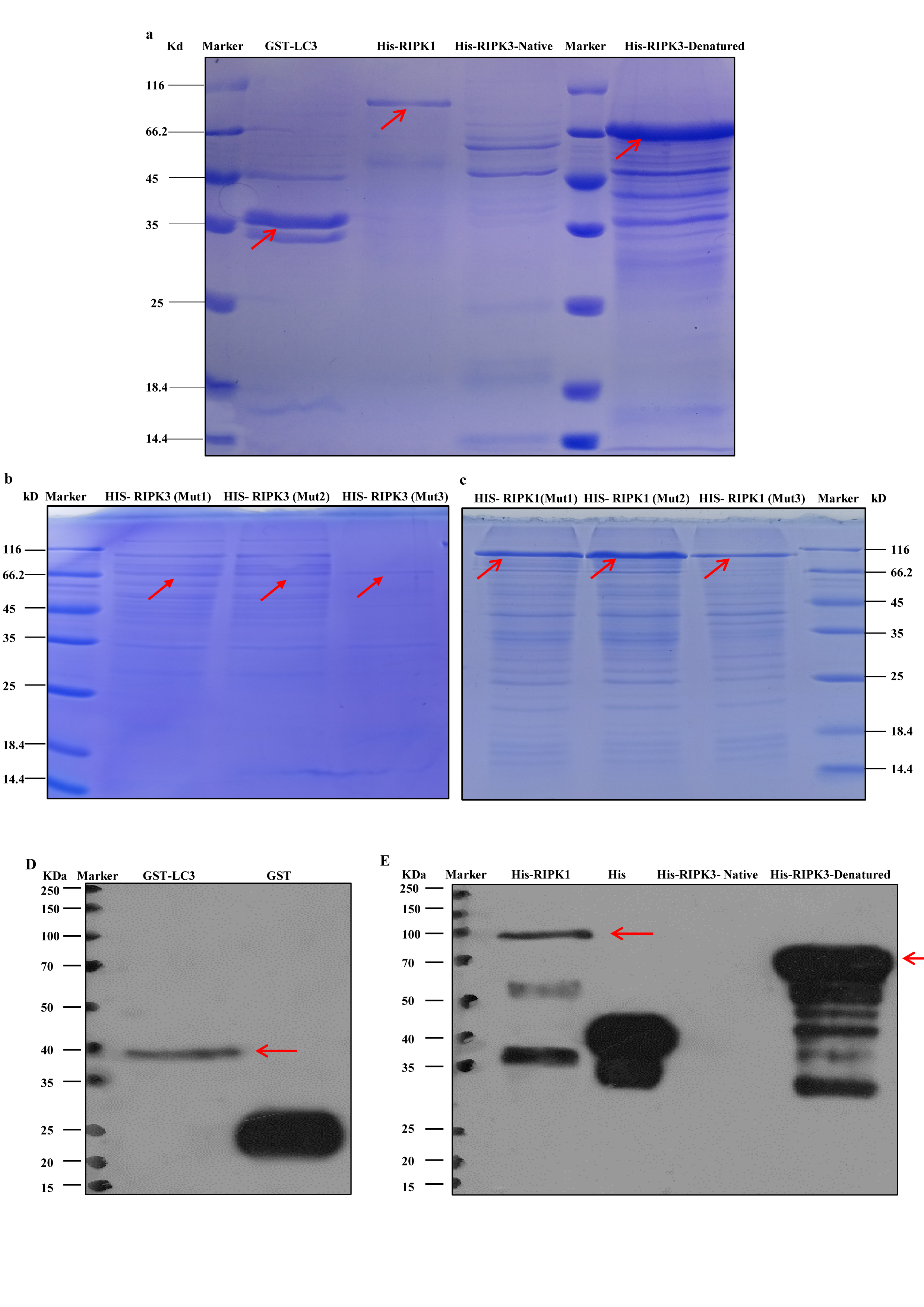

Supplement: Supplementary Figure 2 — (A) GST-LC3, His-RIPK1, and His-RIPK3 were identified through Coomassie brilliant blue staining. Red arrows point to the three proteins. (B) Three mutated RIPK1 protein according to Supplementary Figure 1A were constructed, and identified through Coomassie brilliant blue staining. Red arrows point to the three proteins. (C) Three mutated RIPK3 protein according to Supplementary Figure 1B were constructed, and identified through Coomassie brilliant blue staining. Red arrows point to the three proteins. (D) Immunostaining of GST and GST-LC3. Red arrows point to the protein. (E) Immunostaining of His, His-RIPK1, and His-RIPK3. Red arrows point to the protein. [file Image_2.TIF]

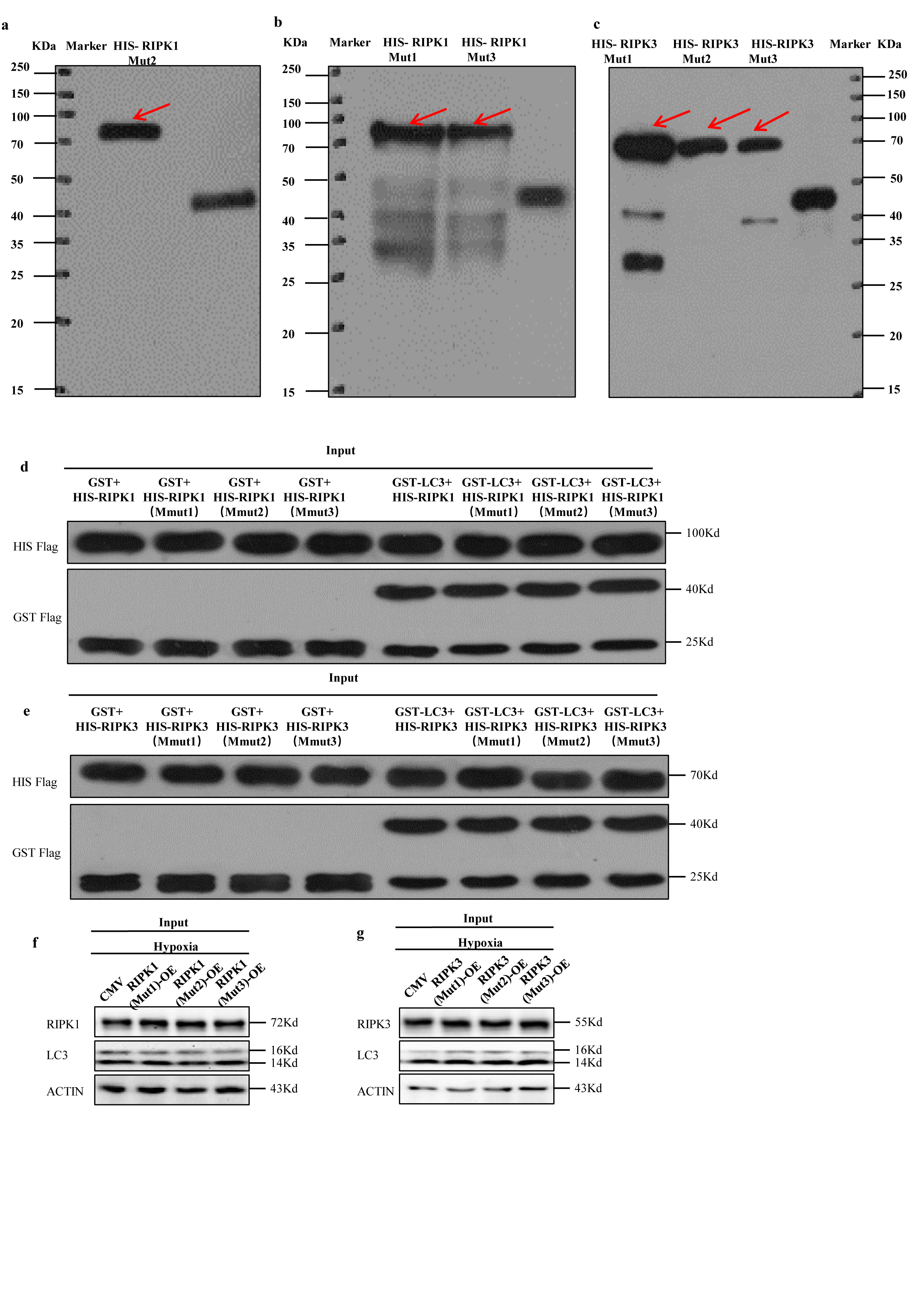

Supplement: Supplementary Figure 3 — (A) Immunostaining of His and His-RIPK1(Mut2). Red arrows point to the protein. (B) Immunostaining of His, His-RIPK1(Mut1), and His-RIPK1(Mut3). Red arrows point to the protein. (C) Immunostaining of His, His-RIPK3(Mut1), His-RIPK3(Mut2), and His-RIPK3(Mut3). Red arrows point to the protein. (D,E) Input of Figure4i and Figure4j, respectively. (F,G) Input of Figure4k and Figure4l, respectively. [file Image_3.TIF]

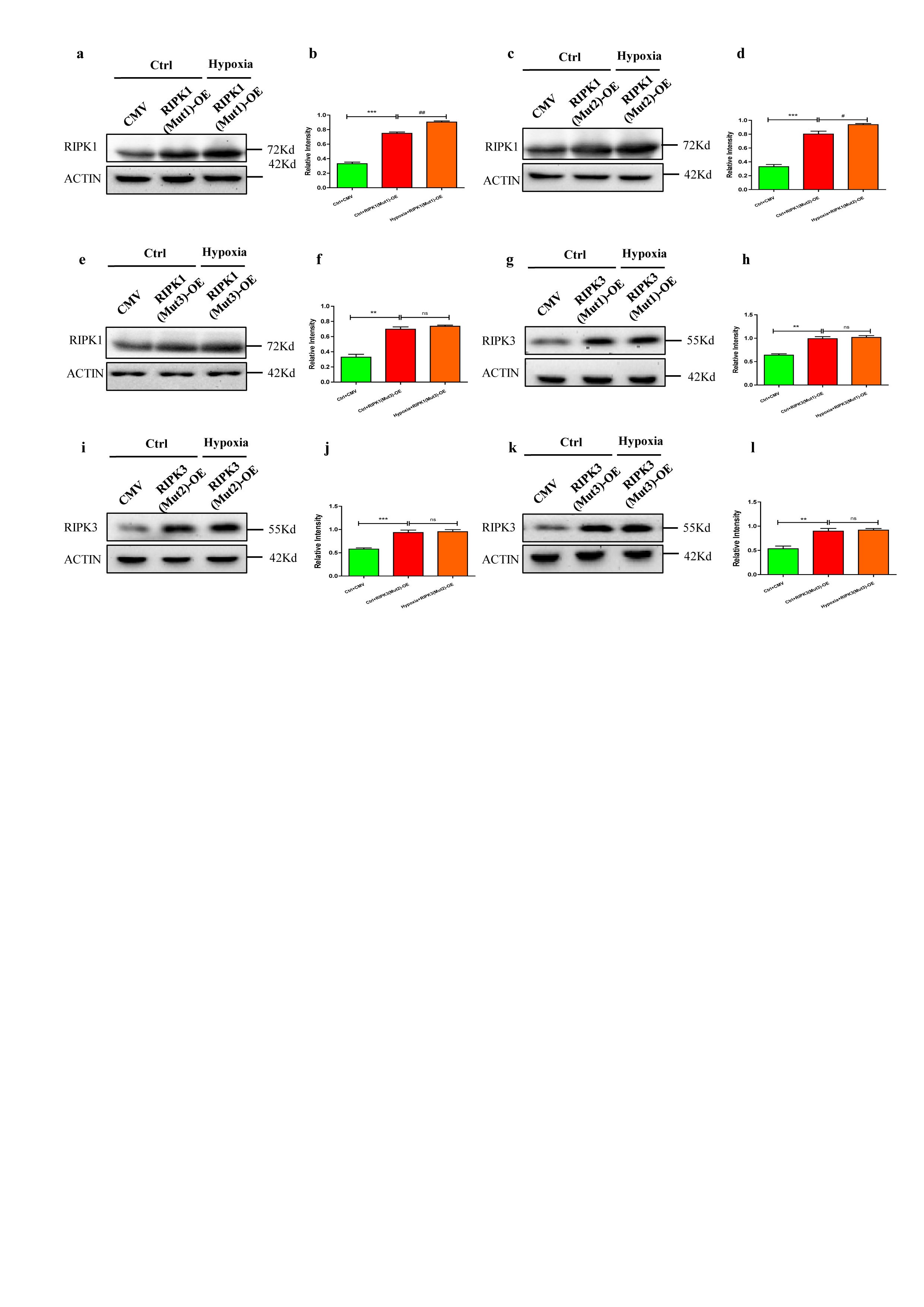

Supplement: Supplementary Figure 4 — (A,B) Western blotting and statistical analysis of RIPK1 from Ctrl+CMV, Ctrl+RIPK1-OE(Mut1) and Hypoxia+RIPK1-OE(Mut1). Mean ± SEM, n = 3. ∗∗∗p < 0.001 versus the Ctrl+CMV group, ##p < 0.01 versus the Ctrl+RIPK1-OE(Mut1) group. (C,D) Western blotting and statistical analysis of RIPK1 from Ctrl+CMV, Ctrl+RIPK1-OE(Mut2) and Hypoxia+RIPK1-OE(Mut2). Mean ± SEM, n = 3. ∗∗∗p < 0.001 versus the Ctrl+CMV group, #p < 0.05 versus the Ctrl+RIPK1-OE(Mut2) group. (E,F) Western blotting and statistical analysis of RIPK1 from Ctrl+CMV, Ctrl+RIPK1-OE(Mut3) and Hypoxia+RIPK1-OE(Mut3). Mean ± SEM, n = 3. ∗∗p < 0.01 versus the Ctrl+CMV group, ns means no statistical difference versus the Ctrl+RIPK1-OE(Mut3) group. (G,H) Western blotting and statistical analysis of RIPK3 from Ctrl+CMV, Ctrl+RIPK3-OE(Mut1), and Hypoxia+RIPK3-OE(Mut1). Mean ± SEM, n = 3. ∗∗p < 0.01 versus the Ctrl+CMV group, ns means no statistical difference versus the Ctrl+RIPK3-OE(Mut1) group. (I,J) Western blotting and statistical analysis of RIPK3 from Ctrl+CMV, Ctrl+RIPK3-OE(Mut2) and Hypoxia+RIPK3-OE(Mut2). Mean ± SEM, n = 3. ∗∗∗p < 0.001 versus the Ctrl+CMV group, ns means no statistical difference versus the Ctrl+RIPK3-OE(Mut2) group. (K,L) Western blotting and statistical analysis of RIPK3 from Ctrl+CMV, Ctrl+RIPK3-OE(Mut3) and Hypoxia+RIPK3-OE(Mut3). Mean ± SEM, n = 3. ∗∗p < 0.01 versus the Ctrl+CMV group, ns means no statistical difference versus the Ctrl+RIPK3-OE(Mut3) group. [file Image_4.TIF]

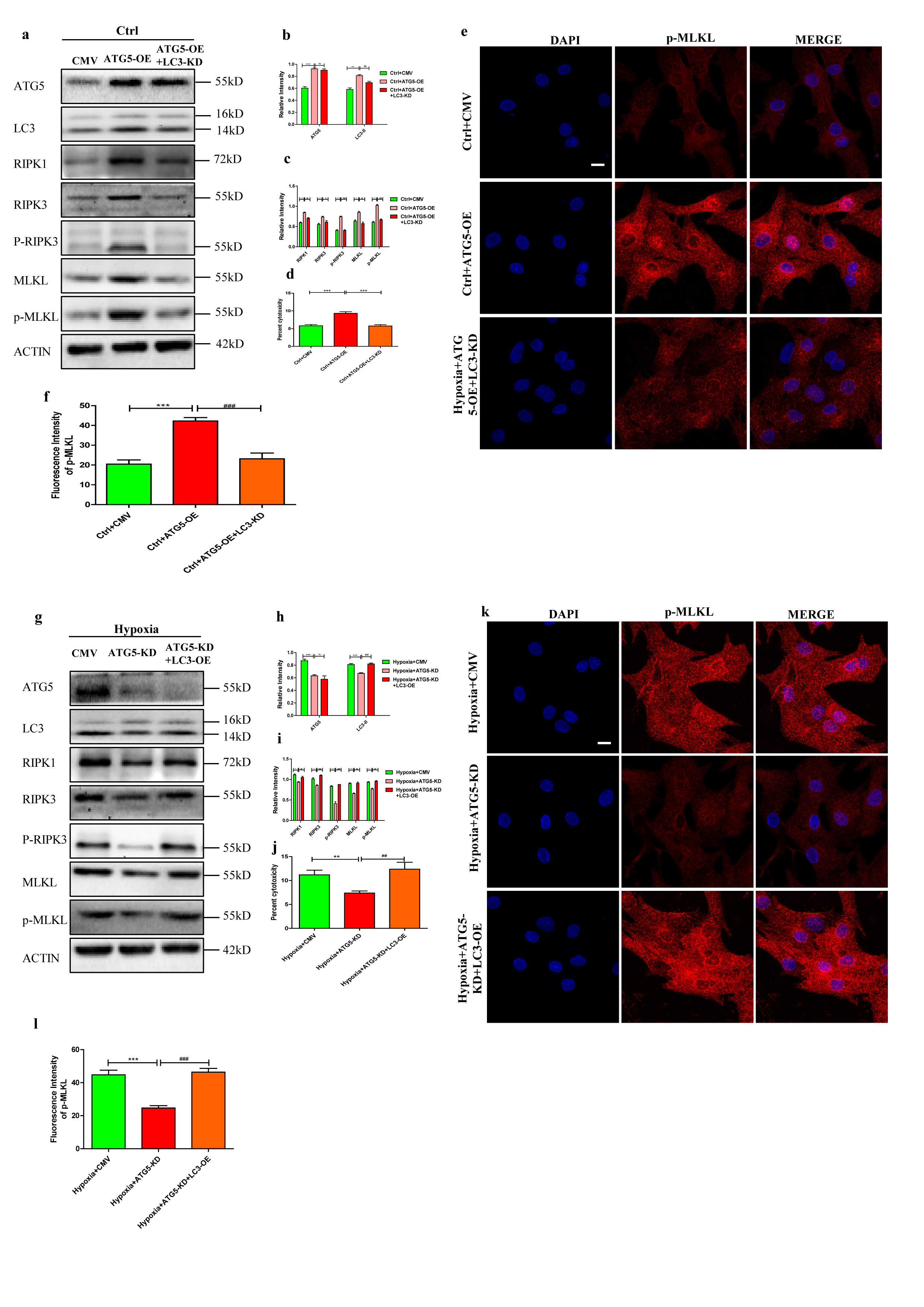

Supplement: Supplementary Figure 5 — (A–C) Western blotting and statistical analysis of ATG5, LC3, RIPK1, RIPK3, p-RIPK3, MLKL and p-MLKL from Ctrl+CMV, Ctrl+ATG5-OE, and Ctrl+ATG5-OE groups. Mean ± SEM, n = 3. ∗p < 0.05, ∗∗p < 0.01, ∗∗∗p < 0.001 versus the Ctrl+CMV group, #p < 0.05 and ##p < 0.01 versus the Ctrl+ATG5-OE group. (D) LDH leakage analysis was performed to determine cell death of corresponding groups in panel (A). Mean ± SEM, n = 3. ∗∗∗p < 0.001 versus the Ctrl+CMV group, ###p < 0.001 versus the Ctrl+ATG5-OE group. (E,F) Representative confocal images and statistical analysis of p-MLKL in corresponding groups in panel (A) Scale bar, 10 μm, Mean ± SEM, n = 3. ∗∗∗p < 0.001 versus the Ctrl+CMV group, ###p < 0.001 versus the Ctrl+ATG5-OE group. (G–I) Western blotting and statistical analysis of ATG5, LC3, RIPK1, RIPK3, p-RIPK3, MLKL, and p-MLKL from Hypoxia+CMV, Hypoxia +ATG5-KD, and Hypoxia +ATG5-KD+LC3-OE groups. Mean ± SEM, n = 3. Ns means no statistical difference, ∗∗p < 0.01, ∗∗∗p < 0.001 versus the Hypoxia+CMV group, ##p < 0.01 and ###p < 0.001 versus the Hypoxia+ ATG5-KD group. (J) LDH leakage analysis was performed to determine cell death of corresponding groups in panel (G). Mean ± SEM, n = 5. ∗∗p < 0.01 versus the Hypoxia+CMV group, ##p < 0.01 versus the Hypoxia +ATG5-KD group. (K,L) Representative confocal images and statistical analysis of p-MLKL in corresponding groups in panel (G) Scale bar, 10 μm, Mean ± SEM, n = 3. ∗∗∗p < 0.001 versus the Hypoxia+CMV group, ###p < 0.001 versus the Hypoxia +ATG5-KD group. [file Image_5.TIF]

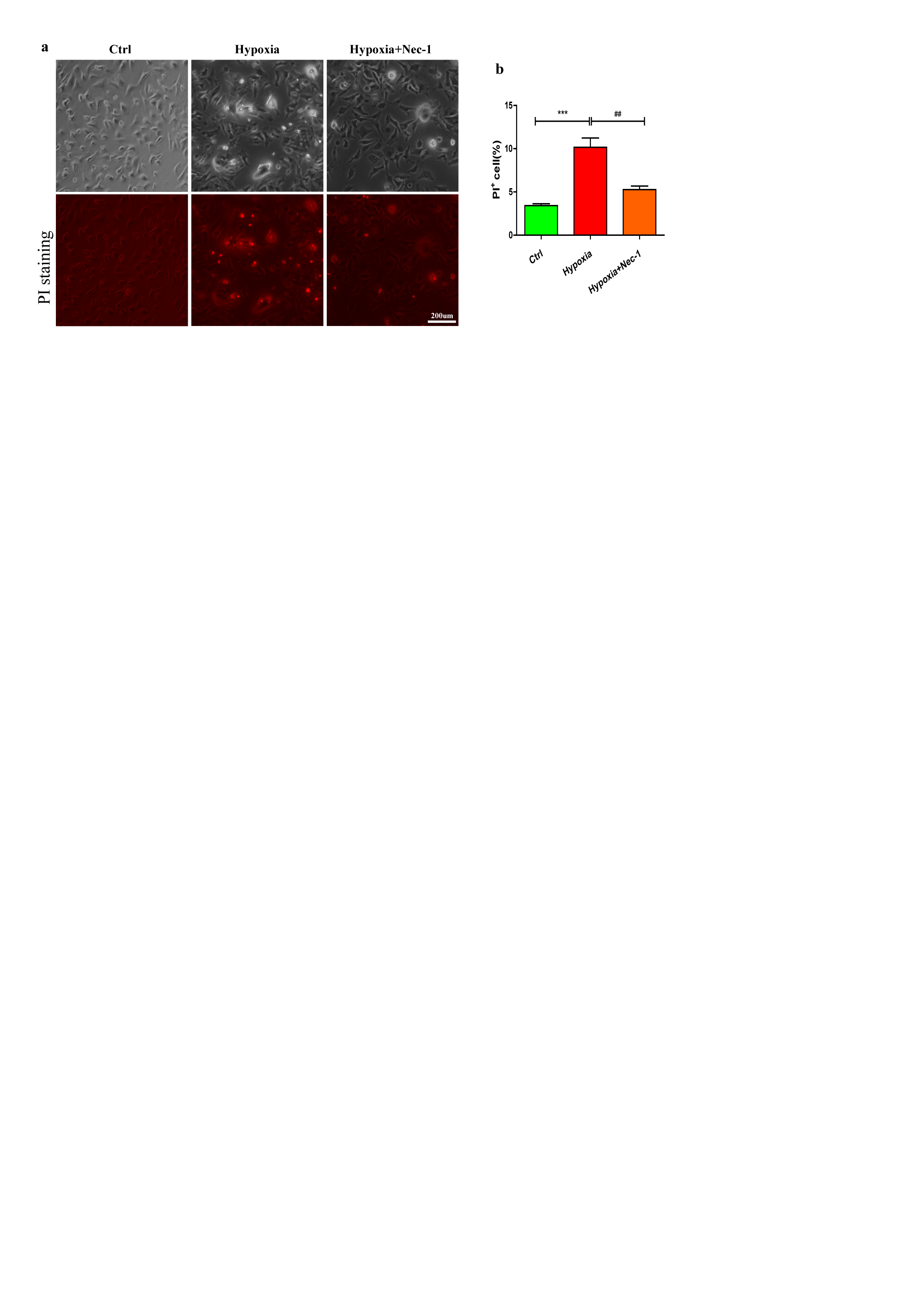

Supplement: Supplementary Figure 6 — (A,B) The PI staining and statistical analysis from Ctrl, Hypoxia, and Hypoxia+Nec-1 groups. Mean ± SEM, n = 3. ∗∗∗p < 0.001 versus the Ctrl group and ##p < 0.01 versus the Hypoxia group. [file Image_6.TIF]
